# Genome assembly of multidrug-resistant *Enterococcus gallinarum* IFEGHNEK1 isolated from bighead carp, *Hypophthalmichthys nobilis*

**DOI:** 10.1128/mra.01063-24

**Published:** 2025-03-20

**Authors:** Basanta Kumar Das, Vikash Kumar, Suvra Roy, Debasmita Mohanty, Souvik Dhar, Smruti Priyambada Pradhan

**Affiliations:** 1 Aquatic Environmental Biotechnology (AEB) Division, ICAR-Central Inland Fisheries Research Institute, Barrackpore, Kolkata, India; Loyola University Chicago, Chicago, Illinois, USA

**Keywords:** *Enterococcus gallinarum*, *Hypophthalmichthys nobilis*, multidrug-resistant, genome sequencing

## Abstract

*Enterococcus gallinarum* IFEGHNEK1 is a multidrug-resistant bacterium belonging to the Enterococcaceae family isolated from bighead carp in 2023. We report the complete genome sequence of *E. gallinarum* (CP169314) containing a 3.15 Mb genome.

## ANNOUNCEMENT

*Enterococcus gallinarum* (family Enterococcaceae) is a gram-positive, catalase-negative, facultatively anaerobic, non-spore-forming cocci (1) that can grow in 6.5% NaCl broth at a wide range of temperatures (from 10℃ to 45°C) (2). *E. gallinarum* IFEGHNEK1 was isolated from bighead carp (*Hypophthalmichthys nobilis*) cultured in Sardar Bherry in the East Kolkata Wetland, West Bengal, India. The symptomatic fish pooled tissue samples (gut, liver, kidney, and skin) were homogenized aseptically and transferred to a conical flask containing sterile tryptone soy broth (TSB) (3). Afterward, based on uniqueness in morphology, size, and color, a single colony was picked from tryptone soy agar plates and cultured overnight in TSB at 28°C. Biochemical assay and 16S rRNA gene sequencing determined by Sanger sequencing before genome sequencing confirmed the presence of the Enterococcaceae family (4).

Bacteria were grown in TSA at 28°C under aerobic conditions for 24 hours. The colonies were scraped, suspended in TE buffer, and pelleted by centrifugation. Genomic DNA was extracted from the pellet using a Qiagen DNeasy PowerSoil Pro Kit (Cat. No: 47014). DNA size distribution (Agilent FEMTO Pulse, Agilent Technologies, USA), DNA quality check (NanoDrop Spectrophotometer and Qubit 3.0 Fluorometer, Thermo Fisher Scientific, USA), DNA shearing (Megaruptor 3, Belgium), Sheared DNA (8–10 kb) and SMRTbell library size distribution (Agilent FEMTO Pulse, Agilent Technologies, USA), SMRTbell library preparation (Template Prepkit 3.0), SMRT library purification, library size selection to remove <5 KB fragments, and preparation of bound complex (Pacific Biosciences, USA) were performed for sequencing. *De novo* whole-genome sequencing was performed on the PacBio Sequel II. The generated raw subreads were converted to HiFi reads using CCS (v6.2.0) (5). HiFi reads provide base-level resolution with 99.9% single-molecule read accuracy. The genome assembly and assessment were done using the Flyev2.9.3, a *de novo* assembler with PacBio-HiFi and plasmid parameters (5). Then, genome completeness was done using the tools “QUAST” and “BUSCO” using bacteria_odb10 as lineage (6, 7). The assembled genomes are complete, circular, and single contig. The ntCard tool is used for genome surveys (8). Later, annotation was done with the Prokaryotic Genome Annotation Process approach of NCBI (v6.8) (9), which combined the alignment-based methods with methods of predicting protein-coding and RNA genes and other functional elements directly from the sequence (10–12). The Resistance Gene Identifier tool uses reference data from the Comprehensive Antibiotic Resistance Database (13). A whole-genome phylogeny was constructed using CLC genomics workbench 12. The k-mer-based tree was built using a neighbor-joining approach, and Mahalanobis’ methods measured the evolutionary distance.

**Fig 1:**
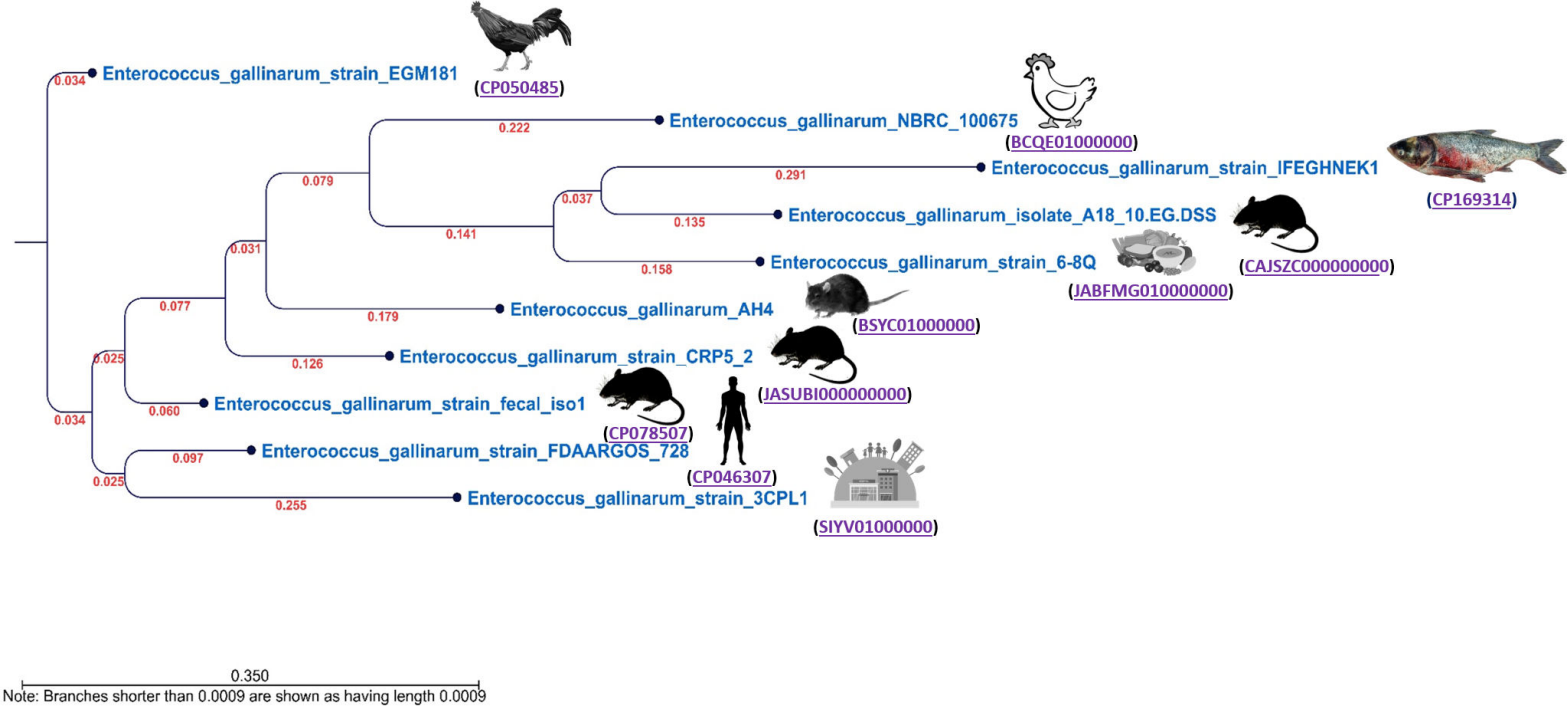
The phylogenetic tree comprises isolated *E. gallinarum* and strains from different sources and countries. This figure was generated using the CLC genomics workbench, and the k-mer-based tree was built using a neighbor-joining approach and Mahalanobis’ methods.

The genomic assembly features of *E. gallinarum* IFEGHNEK1 are presented in Table 1. The genome assembled in this study was 92.44% complete and 0.59% contaminated. It consisted of a closed, circular chromosome of 3150305 bp with a G+C content of 41%, 2,889 protein-coding sequences, 15 rRNA, 61 tRNA, 1 tmRNA, and 2,443 AMR genes. We identified three perfect and two strict AMR genes. The strain was closely related to each other, and they formed a clade with human, mouse, rat, food, hospital waste, and chicken isolates from South Africa, China, Japan, USA, Canada, and Belgium (Fig. 1).

**TABLE 1:** Information on the complete genome sequence of *Enterococcus gallinarum* isolated from fish species

Bacterial isolate	*Enterococcus gallinarum* IFEGHNEK1
Sequencing technology	PacBio Sequel
Assembly method	PacBio Sequel II v. v11.0
HiFi reads	360,387,377
No. of contigs	1
Number of reads	36,017
Total length of contig (bp)	3,150,305
N50	11063
GC%	41
Genome coverage	40.8×
Completeness	92.44%
Contamination	0.59%
Genes	3,020
Protein-coding	2,889
No. of rRNAs	15
No. of tRNAs	61
No. of tmRNAs	1
Antimicrobial resistance genes (total)	2,443 (strict 3, perfect 3, and loose 2,436)
Average nucleotide identity (ANI)	97.72%
Assembly coverage	88.63%
Type assembly coverage	71.01%
BioProject number	PRJNA1152912
Biosample accession no.	SAMN43379292
Accession number	CP169314

## Data Availability

The whole-genome sequence of IFEGHNEK1 is available in GenBank under accession number CP169314. The BioSample and BioProject accession numbers are SAMN43379292 and PRJNA1152912, respectively. The raw sequence data have been deposited in the Sequence Read Archive under accession number SRR30899084.
